# Expression of Concern: The impact of digital economy on high-quality economic development: Research based on the consumption expansion

**DOI:** 10.1371/journal.pone.0331417

**Published:** 2025-09-02

**Authors:** The PLOS One Editors

The *PLOS One* Editors issue an Expression of Concern for this article [1] to notify readers that the article cited in [1] as Reference 50 was retracted after [1] was published due to concerns about potential manipulation of the publication process.

In addition, the *PLOS One* Editors have concerns about the article’s peer review.
