# Supplementary material for: The impact of digital economy on high-quality economic development: Research based on the consumption expansion
Source: PLoS One. 2023 Dec 15;18(12):e0292925. doi: 10.1371/journal.pone.0292925 (PMC10723678; doi:10.1371/journal.pone.0292925)
Supplement: Attachment — Submitted filename: A Rebuttal Letter.docx [file pone.0292925.s002.docx]

Dear academic editor and reviewers,

It is an honor to receive the comments from you. We carefully completed the revision of the manuscript in light of the comments. Specific responses are as follows:

Journal Requirements:

1) Please ensure that your manuscript meets PLOS ONE's style requirements, including those for file naming.

Response: We have revised the full text with reference to PLOS One's format template requirements.

2) We note that the grant information you provided in the ‘Funding Information’ and ‘Financial Disclosure’ sections do not match.

Response: we have ensured the grant information in the system and match them.

3) Please state what role the funders took in the study.

Response: We have stated "The funders had no role in study design, data collection and analysis, decision to publish, or preparation of the manuscript”.

4) We note that you have stated that you will provide repository information for your data at acceptance. Should your manuscript be accepted for publication, we will hold it until you provide the relevant accession numbers or DOIs necessary to access your data. If you wish to make changes to your Data Availability statement, please describe these changes in your cover letter and we will update your Data Availability statement to reflect the information you provide.

Response: We have stated that “The minimal data set underlying this study is placed in the public repository, Open Science Framework and can be accessed at the following link: <http://www.stats.gov.cn/sj/ndsj/>.”

5) We note that all Figures in your submission contain [map/satellite] images which may be copyrighted. All PLOS content is published under the Creative Commons Attribution License (CC BY 4.0), which means that the manuscript, images, and Supporting Information files will be freely available online, and any third party is permitted to access, download, copy, distribute, and use these materials in any way, even commercially, with proper attribution. For these reasons, we cannot publish previously copyrighted maps or satellite images created using proprietary data, such as Google software (Google Maps, Street View, and Earth).

Response: In view of the map copyright issues mentioned by the journal, we have decided to remove the figures from the revised manuscript after careful consideration.

6) Please ensure that you refer to all Figures in your text as, if accepted, production will need this reference to link the reader to the figure.

Response: Not applicable.

7) Please include a separate caption for each figure in your manuscript.

Response: The figures have been removed from the manuscript. This term is not applicable.

**Reviewer #1:**

1) Literature Review: This section is well-aligned and presents an effective blend of recent and past studies. Overall, this section is appropriate in my opinion. Kindly add some studies in literature review section.

Response: After reviewing the references given by the reviewer, we selected some literatures relevant to this study and added citations.

*Page 2, Line 30-31:*

“Many factors including global climate change affect economic development and economic quality [3].

3. Abbass, K., Qasim, M. Z., Song, H., Murshed, M., Mahmood, H., & Younis, I. A review of the global climate change impacts, adaptation, and sustainable mitigation measures. Environmental Science and Pollution Research. 2022; 29(28): 42539-42559. https://doi.org/10.1007/s11356-022-19718-6”

*Page 10, Line 217-218:*

“We construct the index system from the five perspectives of innovation, coordination, green, openness and sharing [49-50].

50. Amjad, A., Abbass, K., Hussain, Y., Khan, F., & Sadiq, S. Effects of the green supply chain management practices on firm performance and sustainable development. Environmental Science and Pollution Research. 2022; 29(44): 66622-66639. https://doi.org/10.1007/s11356-022-19954-w”

2) Study design: This section is well-written and well-explained. Referring to my comments in the Introduction section, try to explain each abbreviation at first and then use its short form.

Response: We checked the full text and added explanations of each abbreviation at first. They were all highlighted in the marked-up copy of our revised manuscript.

3) Empirical Results: Results are explained in detail. Must add much more explanations and interpretations for the results, which are not enough. It is suggested to compare the results of the present research with some similar studies which is done before (more justification is needed).

Response: To make the empirical analysis of this research more reliable, we have added hypothesis 4 in the second part “Theoretical Analysis”.

*Page 8, Line 157-159:*

“Both the development of digital economy and consumption growth will impact on high-quality economic development. This impact path includes both consumer demand and consumption supply.”

*Page 9, Line 177-180:*

“Consequently, hypotheses 3 and 4 were proposed:

H3: Under the regulation of consumption, there is a threshold effect on the impact of the digital economy on high-quality economic development.

H4: Consumption expansion promotes high-quality economic development through the digital economy.”

Also, we revised the models to verify the hypothesis 2 and 4 in the third section “Methodology”.

*Page 9, Line 184-193:*

“Firstly, we constructed the benchmark individual fixed-effect model:

${hqe}_{it}=\alpha_{0}+\alpha_{1}{digit}_{it}+{\alpha_{2}{digit}_{it}^{2}+\alpha}_{3}{control}_{it}+u_{i}+\varepsilon_{it}$	(1)

Where ${hqe}_{it}$ indicates the level of regional high-quality development index of the region $i$ in the period of $t$. $\alpha_{0}$ is the constant. ${digit}_{it}$ is the digital economy index and ${digit}_{it}^{2}$ is the square of ${digit}_{it}$. ${control}_{it}$ represents the control variables. $u_{i}$ indicates individual fixed effects and $\varepsilon_{it}$ is the random error.

The mediational effect model of digit economy is defined as follows.

${hqe}_{it}=\beta_{0}+\beta_{1}{cd}_{it}+\beta_{2}{control}_{it}+u_{i}+\varepsilon_{it}$	(2)

${digit}_{it}=\gamma_{0}+\gamma_{1}{cd}_{it}+\gamma_{2}{control}_{it}+u_{i}+\varepsilon_{it}$	(3)

${hqe}_{it}=\delta_{0}+\delta_{1}{cd}_{it}+{\delta_{2}{digit}_{it}+\delta}_{3}{control}_{it}+u_{i}+\varepsilon_{it}$	(4)”

The results are shown in Table 4.(Page 14)

Mediation effects are further discussed and analyzed in Part IV, section 4.2.

*Page 16, Line 289-321:*

“Mediation effect analysis……

……

It is explained that the consumption supply completely relies on the digit economy to affect the high-quality development.”

In addition, we added discussion to the results of the threshold effect, and analyzed the empirical results of this paper with the existing literature.

*Page 21, Line 369-374:*

” With the increase in consumer demand, the utility of the digital economy to empower high-quality economic development presents an inverted U-shaped structure. In the benchmark model, we verified the inverted U-shaped structure between the digital economy and the high-quality development. Analyzed from the perspective of consumption expansion, the inverted U-shaped structure may be affected by consumer demand. The validation result of hypothesis 2 is strengthened.”

*Page 24, Line 413-424:*

” This result differs from the literature [57]. This may be caused by the samples. The literature analyzed based on 30 cities of China……

……

and the development potential of the digital economy is great, which is most effective in enabling the high-quality development of the regional economy.”

4) Future Directions: Please revise your future research and limitation part into more detail. It would be best if you enhanced your this section.

Response: At the end of the manuscript, we add a paragraph analyzing the limitations and future research directions.

*Page 30, Line 561-566:*

“Finally, this study still has limitations and future directions. The spatial econometric models are not utilized to analyze the spatial impact of digital economy and consumption expansion on high-quality development. In addition, how to measure consumption expansion more comprehensively is also worth exploring. Consumption expansion is only one aspect of consumption growth, the consumption quality and its impact on digital economy and high-quality development also deserves further study. “

**Reviewer #2:**

1) In order to establish the significance and value of the study, it is necessary to provide a comprehensive rationale for the research, which emphasizes its relevance and unique contributions to the current scholarly discourse. This will strengthen the study's originality and scholarly impact.

Response: By combing through the literatures again, the main contributions of this paper are identified in the last paragraph of Part I.

*Page 5, Line 91-97:*

“From the existing researches, we found that consumption expansion is rarely taken into account in the impact of the digital economy on high-quality development. Will the consumption expansion affect digital economy’s impact on promoting high-quality development? And if it does, what is the relationship between them? What about the impact pattern and effect? These questions are all critical, but the conclusions are yet unclear. Clarifying the role of consumption expansion will help to better achieve high-quality economic development.”

2) English check/editing: I found minor typos/ grammatical, this study was presented with depraved English language usage.

Response: We have checked on the spelling and grammar of the full text and invited professionals to help and correct the English editing.

3) The conclusion is sufficiently discussed with some useful policy implications. However, limitations and future directions of research should have a reasonably sized paragraph.

Response: At the end of the manuscript, we add a paragraph analyzing the limitations and future research directions.

*Page 30, Line 561-566:*

“Finally, this study still has limitations and future directions. The spatial econometric models are not utilized to analyze the spatial impact of digital economy and consumption expansion on high-quality development. In addition, how to measure consumption expansion more comprehensively is also worth exploring. Consumption expansion is only one aspect of consumption growth, the consumption quality and its impact on digital economy and high-quality development also deserves further study. “
